# Illness–death model to predict anxiety prevalence in general population during COVID-19 pandemic and beyond: a promising development in mental health epidemiology

**DOI:** 10.1192/bjo.2024.860

**Published:** 2025-03-17

**Authors:** Nathan J. Monk, Ben Beaglehole

**Affiliations:** Department of Psychological Medicine, University of Otago, Christchurch, New Zealand; Department of Māori/Indigenous Health Innovation, University of Otago, Christchurch, New Zealand

**Keywords:** Epidemiology, illness–death model, mental health, prevalence, statistical methodology

## Abstract

Ito et al present an illness–death model projecting 82 scenarios for the prevalence of anxiety disorders in Germany from 2019 to 2030 following the COVID-19 pandemic. We suggest the modelling framework used by Ito et al has promising applications for mental health epidemiology.


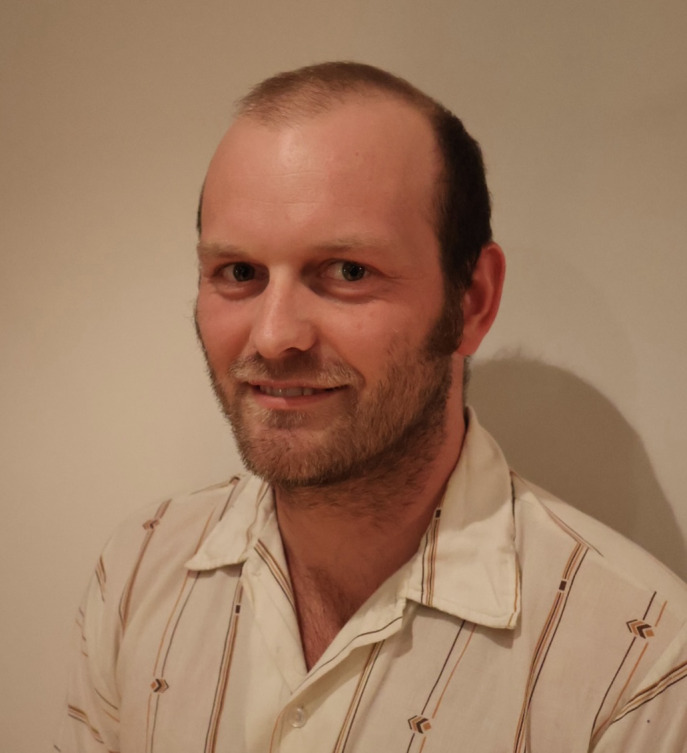
Since its worldwide spread in 2020, COVID-19 has caused considerable physical health morbidity and mortality.^1^ There is also emerging evidence for increased rates of psychiatric conditions such as major depression and anxiety disorders due to the COVID-19 pandemic.^2^

Whether through pathophysiology or social conditions, describing effects of the COVID-19 pandemic on population mental health was promptly identified as a critical research objective.^3^ As a growing body of literature describes the short- and medium-term outcomes of COVID-19 – and unravels some mechanisms through which these effects are occurring^4^ – work in the near future should also consider longer-term projections of increased mental health problems following COVID-19.^3^

Ito et al^5^ pursue this aim: their paper models the prevalence of anxiety disorders in the German population from 2019 to 2030. The authors estimate a series of multistate models (illness–death models) of anxiety disorder in which individuals in the population may transition between three states: susceptible, diseased and dead (see Fig. 1). Note that susceptible and diseased states refer to anxiety disorder being either absent or present, while dead is a state that captures mortality in both people with and without anxiety disorder at the time of death. The authors estimate 82 scenarios for the prevalence of anxiety disorder in the German population from 2019 to 2030. Each iteration of the model varies parameters estimating how pandemic waves during the period 2020–2022 affected transition rates between susceptible, diseased and dead states, and the extent to which the overall effect of the pandemic on anxiety disorder transitions decays over time. The result is a range of anxiety disorder prevalence projections that account for a range of plausible real-world scenarios. Estimates range from small prevalence increases in anxiety disorder (when assuming no impact of the COVID-19 pandemic) to large prevalence increases (when assuming a substantial, slowly decaying impact). Ito et al conclude that their paper demonstrates the feasibility and ease of using the illness–death model to forecast psychopathology prevalence while accounting for waves of the COVID-19 pandemic.

**Fig. 1:**
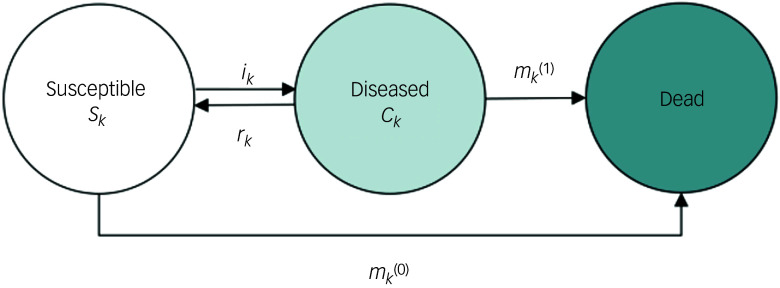
Illness–death model of anxiety disorder prevalence, reproduced from Ito et al.^5^ The population under consideration is divided into three compartments: susceptible (*S_k_*), diseased (*C_k_*) and dead. Arrows between the states indicate possible transitions: incidence rate (*i_k_*), mortality rate among the susceptible (*m_k_*^(0)^), mortality rate among the diseased (*m_k_*^(1)^) and remission probability among the diseased (*r*_*k*_).

As more psychiatric incidence data become available following waves of the COVID-19 pandemic, this will reduce the number of projection scenarios that should be considered. Ito et al^5^ made their projections without knowing the relationship between COVID-19 fluctuations and anxiety disorder incidence – varying assumptions underpinning projection scenarios will be validated (or not) as they correspond to accumulating observations over time (in a similar vein to how, for instance, climate models are appraised).^6^

Illness–death models of chronic disease (e.g. diabetes) require no remission parameter (i.e. an individual cannot transition from having diabetes to being susceptible to diabetes – it is a ‘one-way street’ in that sense). Conversely, Ito et al’s anxiety disorder model requires reciprocity between the susceptible and diseased states: individuals can transition in both directions between these states. The addition of reciprocity to this parameter appears well grounded in Ito et al’s previous work,^7^ and has been performed previously in similar psychiatric prevalence studies using multistate disease modelling.^8^ However, this reciprocity adds another layer of uncertainty and necessitates an increased number of modelling scenarios. Ito et al do not justify their choice of an illness–death model over similar multistate models. It would be possible to specify greater statistical detail using, for instance, a more complex Markov disease progression model (e.g. to capture dimensionality of anxiety). However, a comparatively rudimentary three-state illness–death model appears appropriate for present simulation purposes given (a) the novelty of the analysis, (b) uncertainty in model parameterisation and (c) interpretability and utility of the output in a public health context.

Indeed, there will always be limitations to model parameterisation – ‘All models are wrong, but some are useful’.^9^ Parameter values are informed estimates – but they are estimates nonetheless. For instance, based on one meta-analysis,^10^ Ito et al modelled a mortality risk (i.e. risk of transitioning to dead state) as being 1.4 times higher for the diseased anxiety state than the susceptible anxiety state. However, a more recent meta-analysis suggests no increased mortality risk in anxiety disorder after accounting for publication bias.^11^ This is just one example of the shortfalls of setting parameters based on estimated effect sizes given in the literature. Moreover, many time-dynamic social complexities are infeasible to model. For instance, it is unclear how the COVID-19 pandemic may affect the long-term mental health of children who experienced the pandemic during key developmental years, and will not yet be showing up in mental health prevalence data. Moreover, the economic fallout from the pandemic (e.g. cost of living crisis) will be ongoing, complex and place considerable stress on many people. Representing complex systems in statistical models is non-trivial; as scientists, our best effort in this regard should recruit a combination of biostatistical expertise and content-specific scientific expertise.^12^ In our view, the relatively low number of states and parameters in the illness–death model is a strength at this early stage in the knowledge generation process towards this research objective.

There is obvious public utility in projection modelling of psychiatric prevalences following a mass exposure event such as the COVID-19 pandemic. From a strategy and planning perspective, these projections signal how demand for psychiatric services may fluctuate under specified scenarios. Healthcare systems are overstretched in many countries because population growth is outpacing healthcare resource growth, compounded by the economic impacts of the pandemic on health workforce resourcing and increasing rates of mental health problems. Prevalence projections will not solve these systemic problems, but they will inform planning and strategy to mitigate their stress on health systems and the harm borne by society. If the present approach proves useful in predicting psychiatric prevalence following mass exposure, future work could build on this using more complex Markov modelling to estimate health system demand and economic burden related to prevalence fluctuations.

In the immediate future, we see two threads of research that naturally follow from Ito et al:^5^ (a) projecting other psychiatric conditions and events upon which the pandemic may increase incidence and/or long-term prevalence, and (b) repeating this work in other countries. Anxiety disorders and major depression – high-prevalence conditions that have been linked to the pandemic^2^ – are natural subjects for such modelling. However, other mental health phenomena – particularly those impactful on mental health care demand – should be considered for multistate prevalence modelling. For instance, a decrease in suicide rates was observed in the initial months of the pandemic across many countries.^13^ It is possible that suicide rates will rebound in the wake of waning public health measures and the economic fallout of the pandemic. This situation warrants close monitoring, as it could result in considerable loss of life. Should a spike in suicides be observed in more recent data, illness–death models could model future scenarios based on state transitions informed by historical data.

The COVID-19 pandemic is a ‘once-in-a-century’ global event that has presented an unprecedented opportunity for event-specific research programmes across virtually every social and health science discipline, globally. However, multistate prevalence modelling could also be considered following local- and national-level events. For instance, two large earthquakes in September 2010 and February 2011 caused considerable loss of life and property in Christchurch, New Zealand. The earthquakes and associated challenges resulted in adverse mental health impacts in the Christchurch community across the years immediately following the earthquakes.^14^ Given appropriate local prevalence data, long-term projection modelling of mental health outcomes would be suitable for such events in future to quantify expected stressors of localised events on localised health systems (or of national events on national health systems). Markov multistate modelling has been used to project effects of population mental health interventions^15^; relatively simpler, three-state illness–death models may also have a part to play in future research in this area when simpler parameterisation is preferable.

We see great promise for illness–death models in predicting prevalence following mass exposures in mental health epidemiology. While model parameterisation is, by definition, an imperfect task, the features of the illness–death model appear well suited for projecting long-term psychiatric prevalence while accounting for mass risk and/or protective factors. We have suggested some useful applications that we foresee for this class of model. Additionally, as these models continue to proliferate in the literature, the outcomes they project should be compared with real-world observations as they become available. This should be done to (a) assess overall accuracy and (b) determine specific state transition parameterisations that generate scenarios most akin to real-world observations. A thoughtful, cautious adoption of illness–death models in mental health epidemiology will maximise their potential utility in informing long-term mental health policy and planning following mass exposure events.
